# A Novel Predictor Parameter for Active Recurrent Aphthous Stomatitis: C-Reactive Protein to Albumin Ratio

**DOI:** 10.7759/cureus.5965

**Published:** 2019-10-22

**Authors:** Serkan Kayabasi, Omer Hizli, Serkan Cayir

**Affiliations:** 1 Department of Otolaryngology, Aksaray University, Faculty of Medicine, Aksaray, TUR; 2 Department of Otolaryngology, Giresun University, Prof Dr A. Ilhan Ozdemir Education and Research Hospital, Giresun, TUR; 3 Department of Otolaryngology, Aksaray University, Aksaray Education and Research Hospital, Aksaray, TUR

**Keywords:** aphthous, inflammation, stomatitis, crp, albumin

## Abstract

Objective

Laboratory analysis results may provide an opportunity to predict the activity process of recurrent aphthous stomatitis. The goal of this study was to investigate whether there is a correlation between C-reactive protein to albumin ratio (CAR) and oral ulcer activity in patients with recurrent aphthous stomatitis.

Materials and methods

We included 72 patients (39 with active and 33 with inactive lesion) with recurrent aphthous stomatitis and 60 healthy controls. We compared blood test parameters including CAR, white blood cell count (WBC) and neutrophil to lymphocyte ratio (NLR) among the groups. Additionally, we investigated the most significant parameter for the activity of oral ulcers.

Results

NLR was significantly higher both in the active (p<0.001) and inactive lesion groups (p<0.001), compared to the control group but did not significantly differ between active and inactive lesion groups (p=0.17). A significant difference in median CAR (p<0.001) and WBC (p<0.001) was evident among the three groups. Median WBC was significantly higher in the active lesion group compared to the control group (p<0.001) but did not significantly differ between active and inactive lesion groups (p=0.095). Median CAR was significantly higher in the active lesion group, compared both to the inactive lesion group (p=0.002) and the control group (p<0.001). Median CAR was also significantly higher in the inactive lesion group compared to the control group (p<0.001). Median hemoglobin, platelet to lymphocyte ratio and mean platelet volume did not significantly differ among three groups (p=0.16, p=0.85, p=0.19, respectively).

Conclusion

CAR could be used as a predictive parameter for inflammation and activity of oral ulcers in patients with recurrent aphthous stomatitis.

## Introduction

Recurrent aphthous stomatitis (RAS) is a disease characterized by painful, recurrent, round or oval-shaped ulcers of the oral mucosa. RAS can affect 10-20% of the population [1, 2]. The etiopathogenesis of RAS has not been clearly understood but infection-related, genetic, familial, hormonal, allergic, psychological, traumatic and/or nutritional factors and inflammatory response were suspected [2]. According to the prior literature, histopathological changes indicated an inflammatory condition mainly involving the non-keratinized mucosa [3]. Some triggering factors may have a role in the etiology of RAS or it might simply occur idiopathically [4, 5].

C-reactive protein (CRP) is a positive acute-phase reactant synthesized by the liver and used in the diagnosis of infections and/or evaluation of the effectivity of treatment [6]. Physicians usually use it in the follow-up of an infection or inflammation as a prognostic parameter for the disease because of its short half-life and easy detection feature [6-8]. On the other hand, albumin is a negative acute-phase reactant synthesized by the liver [9]. Albumin level is known to be decreased in acute inflammation, as well as in the ongoing chronic process of inflammation and under poor nutritional conditions [10, 11]. Several prior studies suggested that albumin levels were closely related to inflammation severity, disease prognosis, and mortality [9-11]. In patients with inflammation and infection, CRP levels usually increase, and albumin levels are expected to decrease [12, 13].

C-reactive protein to albumin ratio (CAR) is a novel predictive parameter related to inflammation which is associated with inflammation severity and mortality [14, 15]. Many previous publications reported the higher CAR value as an effective indicator of poor survey in patients with colorectal cancer [16, 17].

In this retrospective, archival and clinical study, we aimed to compare CAR values of patients with recurrent aphthous stomatitis and healthy individuals to determine whether CAR might be a predictive parameter for RAS and activity of lesions. In addition, we investigated the other blood test parameters including neutrophil to lymphocyte ratio (NLR), white blood cell count (WBC), hemoglobin (Hgb), platelet to lymphocyte ratio (PLR) and mean platelet volume (MPV) to determine the most significant predictive parameter for the activity of RAS.

## Materials and methods

Subjects and study design

This retrospective, archival and clinical study was conducted in line with the dictates of the World Medical Association Declaration of Helsinki with the informed consent of the participants and approved by the local ethical committee (IRB number: 2019/06- 24). We searched the medical archive of our institution to identify the cases of recurrent aphthous stomatitis.

We excluded from the study patients with any inflammatory and/or nutritional disease, patients using any medication that might affect the level of blood parameters, patients with any systemic or autoimmune disease, Behçet's disease, pregnancy, acute trauma and the history of malignancy.

We constituted the study groups from the patients with RAS (active lesion group and inactive lesion group) and the control group from healthy individuals. According to the medical history records, patients (previously diagnosed with RAS) with oral ulcer(s) within the previous month were included in the active lesion group and the patients (previously diagnosed with RAS) without any oral ulcer within the previous month were included in the inactive lesion group.

An automated blood cell counter was used for complete blood count measurements (Mindray BC-6000, Shenzhen, China). Serum albumin level was analyzed using automatic photometry commercial kits (Abbott C8000i, Abbott Park, USA). Serum CRP levels were measured using the nephelometric method (AU5800 System, Beckman Coulter Inc, Brea, USA). We noted the blood test parameters including Hgb, WBC, CRP, neutrophil count, lymphocyte count, platelet count, and albumin. Then, we calculated CAR and NLR values of the study groups and the control group. The reference values of CRP and albumin in our hematology laboratory were 0 to 5 mg/dl, and 3.2 to 5.2 g/dl, respectively.

First, we compared the CAR, NLR, WBC, Hgb, PLR and MPV values of the groups. Then, we investigated the most sensitive and specific indicator parameter associated with the activity of aphthous lesions.

Statistical Analysis

Results are presented as mean ± standard deviation for normally distributed data and median (min-max) for abnormally distributed data. We investigated the distribution pattern of the data using the Kolmogorov-Smirnov normality test. The data of age and NLR did distribute normally (p>0.05), thus we used a one-way analysis of variance (ANOVA) test, to compare the mean NLR values of three groups. To analyze the homogeneity of variances, we used Levene’s test (p=0.54), thus we used the Tukey test as the post-hoc test for advanced comparison of NLR. The data of CAR, WBC, Hgb, PLR, and MPV did not distribute normally (p<0.05), thus we used the Kruskal-Wallis test, to compare the median CAR, WBC, Hgb, PLR and MPV values of three groups. For advanced comparisons of CAR, WBC, Hgb, PLR, and MPV, we used the Mann-Whitney U test, as post-hoc test. To detect the most significantly associated parameter with the activity of RAS, and to determine a cut-off value, we used the receiving operator characteristics curve (ROC) analysis test. For statistical analysis of all data, we used SPSS software for Windows (SPSS Inc., Chicago, IL, USA). A p-value less than 0.05 was considered statistically significant. Additionally, for post-hoc comparison tests of abnormally distributed data, we used Bonferroni correction of three groups (triple combination) and a p-value less than 0.017 (0.05/3) was considered statistically significant.

## Results

One hundred thirty-two individuals were eligible for this study. Active lesion group consisted of 39 patients (18 males and 21 females, mean age: 37 ±11 years), inactive lesion group consisted of 33 patients (19 males and 14 females, mean age: 37 ±9 years), and the control group consisted of 60 healthy individuals (28 males and 32 females, mean age: 37 ±11). The groups were age-matched (p=0.98) (Table 1).

**Table 1:** Comparison of demographic data and mean NLR values between groups NLR - neutrophil-to-lymphocyte ratio; M - male; F - female

	Active lesion group ^2^	Inactive lesion group ^1^	Control group^ 0^	p
Mean age (years)	37 ± 11	37 ± 9	37 ± 11	0.98
Gender (M/F)	18/21	19/14	28/32	-
NLR	2.7 ± 0.5	2.5 ± 0.46	1.80 ± 0.43	p^2-0^ <0.001; p^1-0^ <0.001; p^2-1^ = 0.17

The mean NLR value was 2.7 ± 0.5 in active lesion group, 2.5 ± 0.46 in inactive lesion group and 1.80 ± 0.43 in the control group. One-way ANOVA test revealed a significant difference of the mean NLR values among three groups (p<0.001). Post-hoc comparisons revealed that the mean NLR value was significantly higher both in active (p<0.001) and inactive lesion groups (p<0.001), compared to the control group. However, the mean NLR value did not significantly differ between the active and inactive lesion groups (p=0.17). Thus, we found that NLR was not associated with the activity of disease even it was higher in recurrent aphthous stomatitis groups than in the control group.

The median values of CAR, WBC, Hgb, PLR and MPV were presented in the Table 2. In the Kruskal-Wallis test, the median Hgb, PLR and MPV values did not significantly differ among three groups (p=0.16, p=0.85, p=0.19, respectively). However, a significant difference of median CAR (p<0.001) and WBC (p<0.001) was evident among three groups. In post-hoc comparisons, median CAR was significantly higher in the active lesion group, compared both to inactive lesion group (p=0.002) and the control group (p<0.001). The median CAR was also significantly higher in the inactive lesion group compared to the control group (p<0.001). Thus, we found that CAR is a parameter associated both with the recurrent aphthous stomatitis and the activity of disease.

**Table 2:** Comparison of median CRP, WBC, Hbg, PLR and MPV values of the groups * Kruskal-Wallis comparison of three groups CAR - C-reactive protein to albumin ratio; WBC - white blood cell count; Hbg - hemoglobin; PLR - platelet to lymphocyte ratio; MPV - mean platelet volume

	CAR	WBC (K/μL)	Hbg (g/dL)	PLR	MPV (fL)
Active lesion group^2^	1.39 (0.5-5.33)	8.14 (5.6-8.96)	13.96 (12.69-15.44)	129 (80.64-193.51)	8.6 (7.8-10.2)
Inactive lesion group^1^	0.87 (0.47-1.48)	7.38 (6-9.12)	13.96 (11.9-14.22)	127.5 (71.61-169.32)	8.4 (7.7-10.2)
Control group^0^	0.57 (0.32-0.72)	6.12 (5.02-8.98)	13.98 (13.47-14.58)	135.57 (72.99-219.10)	8.3 (7.7-9.8)
p-value*	<0.001	<0.001	0.16	0.85	0.19
P ^2-0^	<0.001	<0.001	-	-	-
P ^1^ ^-0^	<0.001	0.027	-	-	-
P ^2-1^	0.002	0.09	-	-	-

The post-hoc comparisons of WBC revealed that median WBC was significantly higher in the active lesion group compared to the control group (p<0.001). However, median WBC did not significantly differ between the active and inactive lesion groups (p=0.095). Thus, WBC was not associated with the activity of disease.

**Figure 1:**
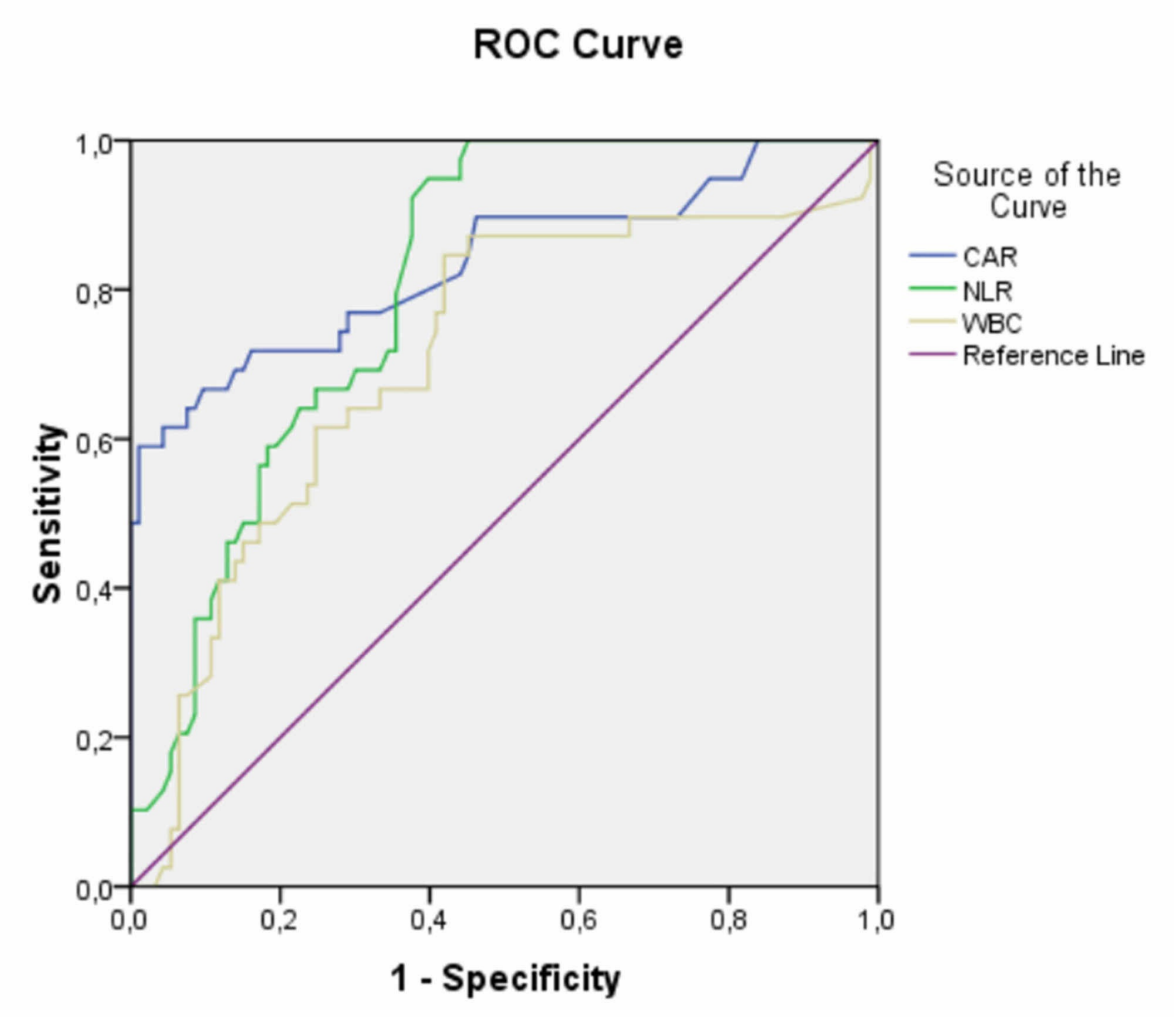
The graph of the ROC analysis ROC - receiver operating characteristic; CAR: C- reactive protein to albumin ratio; NLR - neutrophil to lymphocyte ratio; WBC - white blood cells

Figure 1 represents the graph of the ROC analysis of the parameters included CAR, NLR and WBC. ROC analysis revealed that, CAR had a greater area under curve (0.836) compared both to NLR (0.807) and WBC (0.708) (p<0.001). Thus, CAR was the most associated parameter with the activity of RAS. The cut-off value of CAR for active lesions was found as 0.75, with a sensitivity of 74% and specificity of 72%.

## Discussion

RAS is a mucosal disease affecting 10-20% of the population and can be observed at any age. The etiopathogenesis of RAS has not been completely understood yet. Genetic, immunological, hematological and microbiological factors may contribute to the development of the clinical appearance in RAS [18]. Furthermore, immunological and inflammatory mechanisms may play an important role in the pathogenesis of RAS along with local mucosal trauma, stress and hormonal factors [19]. In previous publications, histopathological changes in RAS showed an inflammatory response involving the non-keratinized mucosa. In addition, increased neutrophil and monocyte concentrations with decreased lymphocyte concentration in the peripheral blood were shown [20].

CRP is a positive acute-phase reactant significantly increasing during infection and/or inflammation, depending on the severity of the disease. Albumin, on the other hand, is a negative acute-phase reactant decreasing in the acute period of infection and/or inflammation [12, 13]. CAR value might have clinical implications in many diseases regarding inflammatory response since overexpression of IL-6 is associated with increased CRP and decreased albumin levels [21].

Wang et al. reported that high CRP and low albumin levels were poor prognostic factors associated with in-hospital mortality in patients with severe acute pancreatitis [22]. Ocal et al. found that CAR was higher in patients with sudden sensorineural hearing loss than in the healthy population [23]. In previous publications, CAR was widely used to predict patient outcomes and surveys in various malignancies including colorectal cancer, esophageal cancer, hepatocellular carcinoma, small cell lung carcinoma and gastric cancer, and in many inflammatory diseases like sepsis, Crohn's disease and acute pancreatitis [14-16, 21-24]. Zhang et al. found that CAR has a significant prognostic value for survival and distant metastasis in nasopharyngeal carcinoma [25]. Additionally, several studies have shown that NLR and PLR were new predictive parameters for the presence of inflammation in RAS. Mean platelet volume (MPV) is another predictive parameter reflecting platelet activity and was shown to be associated with the severity of inflammation [26]. Terzi et al. found that NLR was higher in patients with RAS compared to the control group, but they reported no significant difference of PLR and MPV levels between the patient group and the control group. Ekiz et al. reported higher levels of MPV and erythrocyte sedimentation rate in patients with RAS compared to the healthy population [27]. Ozler et al. reported higher levels of NLR in both active and inactive ulcer periods in patients with RAS compared to the control group [28]. Although many associations of RAS with various blood parameters were focused on in previous publications, the most significant predictive parameter for the activity of RAS lesions is not clear. Our study focused on various blood parameters that might be associated with the disease and activity of the disease, as well as the most significant predictive parameter of active RAS. In our study, PLR and MPV values did not significantly differ among the groups whereas NLR values were higher both in active and inactive lesion groups than in the control group. However, there was no statistically significant difference between NLR values of active ulcer lesion group and inactive ulcer lesion group. Our results suggested that NLR was associated with the presence of RAS rather than the presence of an active lesion.

WBC and its subtypes have been presented as inflammatory markers in various inflammatory diseases [26-28]. In our study, the median WBC was significantly higher in the active lesion group than in the control group. However, no significant difference of WBC was evident between active and inactive lesion groups. In the ROC analysis performed to test the capacity of CAR, WBC, and NLR to show the presence of an active lesion, the area under the curve of CAR was 0.836, whereas of NLR was 0.807 and of WBC was 0.708. Based on these results, CAR was a more significant predictive parameter than NLR and WBC, to determine the activity of RAS. This study also determined the cut-off value of CAR for active lesions as 0.75, with a sensitivity of 74% and specificity of 72%.

The main limitation of our study was the relatively small sample size leading to the lack of generalization. However, to the best of our knowledge, thus far no study in the English language literature has investigated the association between CAR levels and activity of oral ulcers in patients with RAS. In our study, CAR levels were significantly higher in patients with active RAS compared to the inactive RAS group and the control group, indicating the presence of inflammation. CAR might be taken in consideration as a novel potential marker to predict the prognosis in patients with RAS. Nevertheless, further prospective controlled investigations with larger case series focusing on different laboratory tests as a predictor parameter for prognosis are needed.

## Conclusions

CAR is an inexpensive, reproducible, non-invasive, and powerful parameter that can be used as a predictor of inflammation and activity of oral ulcers in patients with RAS. Examination of CAR before initiating a treatment may provide an opportunity to estimate the response to the treatment in RAS patients.
